# Embryonic Origins of Cancer: Insights from Double Homeobox 4 Regulation

**DOI:** 10.3390/biom15050721

**Published:** 2025-05-14

**Authors:** Bo Fu, Hong Ma, Liang Wang, Zhenhua Guo, Fang Wang, Di Liu, Dongjie Zhang

**Affiliations:** 1 Institute of Animal Husbandry, Heilongjiang Academy of Agricultural Sciences, Harbin 150086, China; fubo@haas.cn (B.F.); hongma@haas.cn (H.M.); wangliang@haas.cn (L.W.); guozhenhua@haas.cn (Z.G.); wangfang@haasiah.cn (F.W.); 2 Key Laboratory of Combining Farming and Animal Husbandry, Ministry of Agriculture and Rural Affairs, Harbin 150086, China

**Keywords:** DUX4, zygotic genome activation, embryogenesis, tumorigenesis

## Abstract

Embryogenesis and tumorigenesis share several key biological characteristics, such as rapid cell proliferation, high plasticity, and immune evasion. This similarity indicates that developmental pathways can be hijacked, leading to the formation of malignant cell states. With regard to this, cancer can be regarded as a stem cell disease. On the contrary, a fetus, in many ways, has similar characteristics to the “ideal tumor”, such as immune evasion and rapid growth. Therefore, deciphering the molecular mechanisms beneath these phenomena will help us to understand the embryonic origins of cancer. This review discusses the relationship between embryogenesis and tumorigenesis, highlighting the potential roles played by DUX4. DUX4 is involved in the activation of the zygote genome and then facilitates the establishment of totipotency in pre-implantation embryos, whereas the misexpression of DUX4 is associated with different types of cancer. Taken together, this indicates that DUX4 performs analogous functions in these two processes and connects embryogenesis and tumorigenesis. Through examining DUX4, this review underscores the importance of developmental mechanisms in cancer biology, suggesting that the insights gained from studying embryonic processes may provide novel therapeutic strategies. As we continue to explore the complex relationship between cancer and embryogenesis, elucidating the role of DUX4 in linking these two processes will be critical for developing targeted therapies that exploit developmental pathways.

## 1. Introduction

Double Homeobox 4 (DUX4), a member of the Dux gene family, has only been identified in mammals to date. DUX4 is a transcription factor that plays an important role in early embryonic development and pathological conditions, particularly facioscapulohumeral muscular dystrophy (FSHD) and several types of cancer. It primarily functions in activating the zygotic genome and initiating transcription during the early stages of embryogenesis [1,2]. The macrosatellite repeat sequence of D4Z4 on human chromosome 4q35 encodes DUX4, and the epigenetic mechanism strictly regulates DUX4 expression. In early embryos, chromatin remodeling and the generally permitted state of transcription are caused by chromatin relaxation and increased histone fluidity, which may increase the availability of DUX4’s promoter regions [3,4]. In healthy adult tissues, the D4Z4 region is heavily methylated to suppress DUX4 activation. In addition to DNA methylation, repressive histone marks, such as H3K9me3 and H3K27me3, are also found at the DUX4 locus in somatic tissues, and these modifications create a heterochromatin-like structure, which limits access to the DUX4 promoter and prevents its transcriptional activation [5].

Under pathological conditions, DNA hypomethylation at the D4Z4 repeat region of chromosome 4q35 leads to the derepression of DUX4. This derepression can trigger the toxic effects observed in muscle cells [6] and is associated with several diseases, most notably FSHD, where a deletion in the D4Z4 region leads to the reactivation of DUX4 expression. This aberrant expression subsequently contributes to muscle degeneration [7]. DUX4’s misexpression is also linked to cancer, particularly sarcomas and B-cell leukemia, where it plays a role in cell proliferation, apoptosis, immune evasion, and transcriptional deregulation [8].

The DUX4 structure comprises two domains of homologous form. These domains mediate the protein’s key DNA-binding activity and regulation of target gene expression. The crystal structure of the homeodomain in complex with DNA reveals how DUX4 recognizes specific DNA sequences [9]. Mechanically, DUX4 binds P300/CBP to the target gene to stimulate the acetylation of H3K27, which simultaneously leads to nucleosome rearrangement, thus increasing chromatin availability and activating gene expression. Changes in *Zscan4* site acetylation and Histone H3 depletion, as well as Chip-seq data, support its role in chromatin remodeling and nucleosome rearrangement [10]. Subsequently, DUX4 activates a shared set of genes, including ZSCAN4, TRIM, PRAMEF, and retrotransposon-associated elements, which are expressed in both early embryos and tumor cells, thereby inducing a metastable, totipotent-like state and enhancing immune evasion and cellular plasticity.

It is well established that cancer cells and early embryos share several biological features, including rapid proliferation, high plasticity, and invasive behavior, which are essential for both tumor progression and embryogenesis [11]. Furthermore, both cell types exhibit altered epigenetic regulation, metabolic shifts, and stem cell-like properties, which contribute to their ability to proliferate, migrate, and resist differentiation, promoting cancer progression or development [12,13,14,15]. From a certain perspective, cancer can be seen as an evolutionary reversion phenomenon, where malignant cells reactivate developmental programs and adopt a primitive, stem-like phenotype reminiscent of early embryogenesis or unicellular organisms. This enables them to proliferate uncontrollably, resist differentiation, and adapt to selective pressures [16]. With growing insights into the functions of the DUX4 gene and its regulatory mechanisms, it is now evident that DUX4 plays a pivotal role in both embryogenesis and oncogenesis, acting as a molecular bridge between these two processes. This conceptual framework provides a new perspective for identifying developmentally inspired vulnerabilities in cancer, potentially paving the way for innovative therapeutic strategies.

This review aims to offer an overview of the pivotal role of DUX4 in early embryonic development and tumorigenesis, emphasizing the parallel functions it fulfills in both processes. Elucidating how DUX4 mediates embryonic gene activation and tumor progression will shed light on the mechanisms by which cancer cells hijack developmental pathways to promote proliferation and metastasis. This may offer fresh perspectives on how embryonic mechanisms might be leveraged to tackle diseases and provide promising mechanism-based cancer treatments.

## 2. The Intersection of Embryogenesis and Tumorigenesis

As early as the 19th century, researchers have suggested that cancer cells may originate from cells that have returned to a more embryonic or undifferentiated state, laying the foundation for modern cancer biology [13,17,18,19]. Modern research has confirmed that some of the provisions of the early hypothesis are still true. In many malignant tumors, researchers have observed cellular reprogramming phenomena that parallel the plasticity states characteristic of embryonic development. This cellular reprogramming phenomenon is now recognized as a common feature across diverse cancer types. In 1829, gynecologic surgeon Joseph Récamier observed small cells in the early stages of development in tumors and hypothesized that some types of cancer could arise from primitive germ cells. This concept was formalized in the “embryonic rest theory”, which Francesco Durante generalized in 1874. According to this theory, tumors originate from cells that either retain or reacquire embryonic characteristics [17], specifically from undifferentiated embryonic cells persisting in adult tissues or differentiated adult cells that undergo dedifferentiation to an embryonic-like state. The concept has caused controversy in stem cell research and dedifferentiated cancer [20]. Concurrently, British evolutionary biologist John Beard observed significant morphological and behavioral similarities between trophoblast cells and invasive tumors, and the basic principle of acquiring the invasive properties of cancer cells necessary for metastases is reflected in the modern understanding of the metastatic cascade [21].

The intersection between embryogenesis and tumorigenesis reveals striking molecular and cellular parallels, and embryogenesis and tumorigenesis share fundamental similarities in rapid cellular proliferation, cell competition mechanisms, stem cell properties, dedifferentiation patterns, cellular plasticity, complex intercellular signaling pathways, immune evasion strategies, epigenetic flexibility through bivalent chromatin marks, and retrotransposon activation. In detail, both processes exhibit rapid cellular proliferation, though cancer disrupts the size control mechanisms that regulate normal tissue growth through mutations in oncogenes and tumor suppressor genes [11,22]. Cell competition, an evolutionarily conserved mechanism controlling embryonic tissue size, becomes significant in cancer biology, as mutations in oncogenes enhance competitive interactions that facilitate tumor growth [23,24,25]. The microenvironment also plays a crucial role in both contexts, as demonstrated by teratoma formation studies highlighting the importance of both “seeds” (stem cells) and “soil” (microenvironment) in tumorigenesis [26,27,28]. Cellular differentiation status significantly impacts tumor classification and prognosis, exemplified by cases like acute promyelocytic leukemia, where dedifferentiation can be therapeutically targeted [29,30,31]. Cellular plasticity, particularly in epithelial–mesenchymal transition (EMT) and mesenchymal–epithelial transition (MET), facilitates both embryonic organ formation and cancer metastasis [32,33,34]. Complex cellular crosstalk characterizes both embryonic induction and tumor progression, with cancer cells functioning as “tumor organizers” that dictate the composition of their microenvironment, similar to embryonic signaling centers like the Spemann–Mangold organizer [35]. Both embryonic and cancer cells manipulate immune responses through mechanisms, including immune checkpoint protein expression (PD-L1), secretion of immunosuppressive factors (TGF-β), and creation of immunosuppressive microenvironments [36,37,38]. Epigenetic plasticity manifests in both contexts through shared mechanisms like bivalent chromatin marks (H3K4me3 and H3K27me3) [39,40]. Additionally, both cell types demonstrate the activation of retrotransposons, such as LINE-1 and endogenous retroviruses, which influence cellular reprogramming, proliferation, and differentiation in embryogenesis [41,42] while contributing to genomic instability and the disruption of tumor suppressor genes in cancer [43,44]. All these similarities indicate that cancer frequently hijacks developmental programs, utilizing embryonic cellular mechanisms within an aberrant context.

Of particular importance, tumorigenesis has also been shown to mimic pathways essential for embryonic development, which indicates that the genetic origins of these pathways in tumorigenesis may be similar to those of embryonic development. During this process, transcription factors play a crucial role. Particularly, DUX4, a transcription factor that is specifically expressed during early embryonic development, is highly expressed in cancer cell lines. The functional importance of DUX4 lies in its ability to regulate zygotic genome activation by directly activating numerous genes expressed during ZGA. However, recent research has identified cancer cell lines that express DUX4 and demonstrated that this expression not only activates early embryonic programs but also suppresses steady-state and interferon-induced MHC class I expression, which indicates that the expression of DUX4 in cancer cells may have implications for cancer growth, progression, and immune evasion [45].

**Figure 1:**
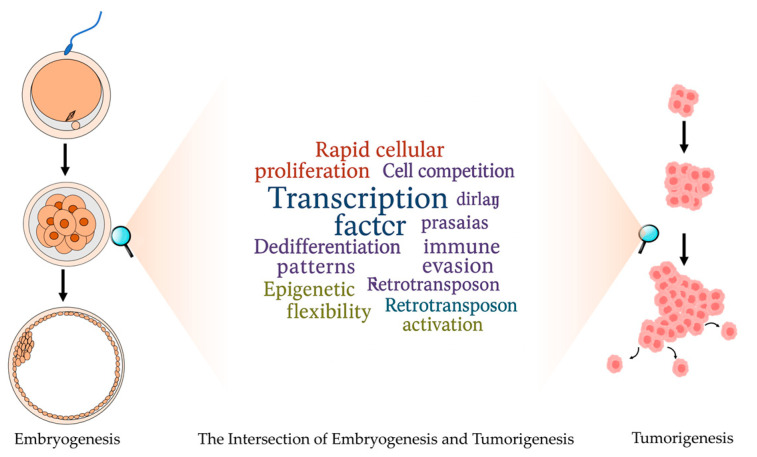
The intersection between embryogenesis and tumorigenesis. On the left, embryonic development is depicted, focusing on key processes such as fertilization, cleavage, and blastocyst formation. On the right, tumorigenesis is illustrated, emphasizing key processes, including tumor initiation, progression, and metastasis. The central word cloud has shown that embryogenesis and tumorigenesis share several key biological processes, such as rapid cell proliferation, high plasticity, immune evasion, and so on.

Taken together, embryogenesis and tumorigenesis share key molecular and cellular processes, which facilitate both tumor progression and normal development. The diagram, shown in Figure 1, illustrates this intersection between embryogenesis and cancer development.

## 3. The Role of DUX4 in Early Embryonic Development

ZGA is a critical event in early embryonic development, marking the transition from maternal control of gene expression to the activation of the embryonic genome. With regard to human embryos, ZGA begins during the four-cell to eight-cell stage, when the major transcriptional activation occurs. This timing is a crucial milestone, as it lays the foundation for subsequent cell differentiation and development [46]. ZGA is orchestrated by a complex network of transcription factors. Among these factors, DUX4 plays a pivotal role in initiating the expression of genes involved in early developmental pathways. DUX4 is expressed prior to ZGA in human embryos and interacts with the p300/CBP complex to promote chromatin relaxation, ultimately facilitating the activation of ZGA genes. It achieves this by recruiting the histone acetyltransferases p300/CBP through its C-terminal domain. DUX4 binding was specifically associated with pronounced acetylation changes at the ZSCAN4 locus, notably characterized by an increased enrichment of H3K27ac flanking the DUX4 binding region. This observation suggests a reorganization of nucleosome positioning at these loci. Additionally, DUX4 demonstrates the capacity to bind nucleosome-occupied DNA and promote chromatin remodeling. This is supported by evidence of nucleosome displacement, especially at sites that were previously inaccessible subsequent to DUX4 binding. ChIP-seq data further indicate that DUX4 binding correlates with histone H3 depletion and H3K27ac enrichment at target loci, supporting a functional role in nucleosome repositioning [10].

Its molecular function involves directly binding to promoters and regulatory regions of early embryonic genes and retrotransposons such as HERV-L (Human Endogenous Retrovirus-L), triggering their transcription, which is critical for transitioning from maternal to zygotic control of gene expression during the first few cleavage stages in human embryos [47]. In detail, DUX4 binds to specific DNA motifs in the promoters of ZGA-associated genes, such as ZSCAN4, LEUTX, KDM4E, and PRAMEF family genes. Its homeodomains interact with major and minor grooves of DNA, promoting chromatin remodeling and gene transcriptional activation, which is essential for totipotency and cleavage-stage identity in early embryos [48]. DUX4 also binds to the Long Terminal Repeats (LTRs) of HERV-L, thereby initiating their transcription. HERV-L (Human Endogenous Retrovirus-L) is a family of retroelements that are reactivated during early embryonic development, specifically during the ZGA phase. DUX4 directly activates HERV-L retrotransposons, which are integral in establishing the transcriptional program required for totipotency in early embryonic cells. This activation is critical for the proper reprogramming of the genome in early-stage embryos and contributes to the transcription of adjacent or embedded ZGA-related genes through enhancer-like effects [49,50]. ATAC-seq data reveal increased chromatin accessibility at DUX4-binding sites following DUX4 expression, which indicates that DUX4 modifies the chromatin structure to create a permissive transcriptional environment and then acts upstream in the transcriptional hierarchy of ZGA [51].

In vitro models like eight-cell-like cells (8CLCs) demonstrate that the overexpression of DUX4 also leads to the induction of ZGA transcriptional programs and HERV-L elements, and then 8CLCs models recapitulate the transcriptional landscape of the eight-cell human embryo. Eight-cell-like cells are derived from naive human pluripotent stem cells. These cells were cultured under conditions that favored their transition into a state that closely resembled that of the human eight-cell embryo. The key markers of this transition were tracked using specific reporter genes and cellular markers, such as TPRX1 and H3.Y, which are characteristic of the eight-cell stage in human embryos. To characterize the transcriptional profile of 8CLCs, Taubenschmid et al. performed RNA sequencing to compare the transcriptome of 8CLCs with that of the human eight-cell embryo. Intriguingly, they observed the expression of ZGA markers (e.g., ZSCAN4 and LEUTX) and the activation of HERV-L retrotransposons, both of which are key features of zygotic genome activation in early embryos. This provided evidence that 8CLCs have a transcriptional landscape similar to that of eight-cell embryos during ZGA, and DUX4 is sufficient to induce ZGA [50]. Notably, although human DUX4 interacts with ERV elements and activates genes that drive cleavage-stage transcriptional programs, this effect may not be universally conserved across species, as seen in some mouse models. The divergence in the regulatory domains of DUX4 across species, especially with regard to ERV binding, reveals that while DUX4 maintains a conserved role in activating cleavage-stage genes, its interaction with retrotransposon promoters is more specialized in humans. In other words, human DUX4 was able to activate genes in mouse cells driven by conventional promoters, which are typically involved in early embryonic transcription; however, it failed to activate retrotransposons in mouse cells, such as MERVL. This phenomenon confirmed that human DUX4 can activate embryonic genes in mouse cells, but the activation of retrotransposon sequences by DUX4 exhibits species specificity [49].

## 4. The Role of DUX4 in Tumorigenesis

DUX4 is typically repressed in somatic tissues, but its expression becomes dysregulated in various pathological states. Although the aberrant expression of DUX4 has been predominantly studied in the context of FSHD, recent studies have demonstrated that DUX4 is ectopically expressed in various cancers and plays a critical role in tumor progression, immune evasion, and therapy resistance. There has been shown to be abnormal DUX4 expression and suppression of the immune response in melanoma and bladder, breast, and prostate cancers. This immune response is considered one of the key factors in the resistance of tumors with high DUX4 expression to treatment [52]. Abnormal expression of DUX4 has been observed in sarcoma and hematologic malignancies, which indicates that DUX4 may function as an oncogene. Translocation of DUX4 is associated with B-cell leukemia. Studies have shown that the overexpression of DUK4 in these malignancies can destroy normal cell differentiation pathways and promote malignant transformation [9].

The mechanisms leading to DUX4 activation in malignancies are diverse. The abnormal expression of DUX4 in cancers, particularly sarcomas and leukemias, results from both genetic alterations (such as translocations) and epigenetic reprogramming. This expression can be controlled through complex interactions involving DNA methylation, histone modifications, and noncoding RNAs [5]. In addition, when herpesviruses infect host cells, they can trigger the re-expression of DUX4 as part of the virus’s strategy to overcome host immune defenses and support viral replication. In detail, herpesviruses activate DUX4 expression through their immediate-early proteins ICP0 and ICP4, where ICP4 binds directly to the DUX4 promoter and ICP0 facilitates chromatin derepression through its E3 ubiquitin ligase activity, which, in turn, activates hundreds of embryonic-like target genes and retroelements, ultimately creating a permissive chromatin environment that facilitates viral gene transcription and replication [53].

Abnormal expression of DUX4 in somatic cells activates the expression programs of embryonic genes, thus affecting the processes of proliferation, invasion, metastasis, and immune evasion and gradually contributing to the formation of tumors. Studies of myoblast precursor cells have shown that DUX4 can regulate the cell cycle and genes associated with proliferation, inhibit cell differentiation, and maintain a proliferative state. This high proliferative activity is associated with the state of stem cell phenotype and tumorigenesis [54]. In sarcomas, fusion proteins such as CIC-DUX4 promote malignant development by regulating pathways such as ETV4, which is required for cancer cell invasion and metastasis [55]. In sarcomas, fusion proteins such as CIC-DUX4 promote malignant development by regulating pathways such as ETV4, and ETV4 is necessary for the invasion of cancer cells and metastasis [55]. CIC-DUX4 fusion leads to the activation of molecular networks involved in tumor growth and metastasis and activates the transcription factor ETV4 [56]. Expression of CIC-DUX4 in zebrafish models induces small round blue cell tumors, recapitulating the characteristics of human CIC-DUX4 sarcomas. This model provides valuable insights into tumor formation and metastasis, with ETV4 again being identified as a central driver of metastasis [57]. In cancer contexts, DUX4 has also been shown to suppress MHC class I expression, contributing to immune evasion and possibly promoting tumor proliferation by reducing anti-tumor immune responses. Mechanistically, DUX4 suppresses MHC-I expression through multiple mechanisms, thereby promoting immune evasion in tumor cells. Firstly, DUX4 inhibits interferon-γ-induced MHC-I gene expression, hindering antigen presentation. Secondly, DUX4 interferes with the function of the transcription factor STAT1, suppressing MHC-I transcriptional activation. Additionally, DUX4 regulates the phosphorylation status of translation initiation factors such as eIF2α and eIF4E, inhibiting MHC-I protein translation. These combined mechanisms lead to a reduction of MHC-I molecules on the tumor cell surface, decreasing the immune system’s ability to recognize and eliminate tumor cells. This evasion mechanism helps cancer cells to survive and proliferate in hostile tumor microenvironments, highlighting DUX4’s role in supporting cancer cell growth [52]. DUX4 interacts with several oncogenic pathways, including p53, WNT, and MYC, playing a significant role in both the pathogenesis of FSHD and various cancers. Specifically, DUX4 modulates WNT/β-catenin signaling in FSHD, promotes tumorigenesis via MYC pathways in rhabdomyosarcoma, and influences MYC amplification in CIC-DUX sarcomas while also exhibiting p53-independent toxicity [58,59,60,61].

## 5. The Analogous Function of DUX4 Between Embryogenesis and Oncogenesis

DUX4 functions as a crucial transcription factor involved in early embryogenesis, but its impact on cell proliferation extends beyond development and plays an essential role in various diseases, particularly in muscle and cancer pathologies. In FSHD, aberrant expression of DUX4 in muscle cells disrupts normal differentiation, leading to the formation of atrophic, disorganized muscle fibers and severe muscle weakness [62]. DUX4 expression promotes the activation of various pathways involved in cell cycle progression, which leads to increased cell proliferation. This unregulated proliferation, while essential in embryonic development, can be pathological when it occurs in somatic cells. Specifically, DUX4 can activate proliferative pathways such as the IGF-1 and PTEN signaling pathways, contributing to the uncontrolled cell division seen in FSHD [63]. This DUX4-induced excessive or deregulated proliferation also contributes to tumorigenesis, which is seen in the context of cancer. In cancer, the ability of DUX4 to promote cell proliferation at the expense of differentiation has been implicated in tumor progression. Misexpression of DUX4 has been linked to several types of cancer, including sarcomas and leukemias, where it suppresses immune responses and contributes to tumor growth.

In the context of oncogenesis, DUX4’s expression in cancer cells has been shown to contribute to immune evasion, a mechanism that allows tumors to avoid detection by the host’s immune system. One of the primary ways DUX4 achieves immune evasion is by suppressing the expression of major histocompatibility complex (MHC) class I molecules, which are essential for the immune system to recognize and attack cancer cells. This suppression occurs through the interaction of DUX4 with STAT1, which is a key regulator of immune response pathways. DUX4 effectively blocks the interferon-γ (IFNγ)-induced activation of MHC class I expression [64]. As DUX4 hinders MHC class I-mediated antigen presentation, which is crucial for the activation of CD8+ T cells, and downregulates the expression of genes involved in immune cell recruitment to the tumor site, this suppression leads to immune exclusion, where immune cells, particularly T cells, are unable to infiltrate the tumor [65]. In metastatic cancers, DUX4 expression has been found to correlate with a reduced response to immune checkpoint inhibitors, such as the PD-L1 blockade, highlighting its role in immunotherapy resistance [65]. During embryogenesis, cells also evade immune surveillance. This mechanism supports their persistence and development, mirroring the immune evasion strategies observed in cancer cells. This phenomenon, often referred to as “immune tolerance” or “immune privilege”, has significant biological implications for both the embryo and the maternal organism. The maternal immune system is designed to protect the body from foreign pathogens and is capable of recognizing non-self antigens, such as those introduced by pathogens or foreign tissues, including paternal antigens present in the embryo. However, the immune system must adapt to prevent attacking the embryo, which contains antigens from both the mother and the father [52]. In early embryos, the expression of DUX4, an inhibitor of MHC class I, may reduce the likelihood that maternal immune cells recognize the embryo as foreign and attack it [52].

DUX4 activation is involved in the transcription of genes associated with ZGA. This process marks the transition from maternal genome control to zygotic genome control, which is necessary for normal development. This mechanism is also co-opted by cancer cells. The reactivation of DUX4-associated genes in cancer may contribute to tumor progression. *Zscan4*, a target gene of DUX4, is highly expressed in two-cell stage embryos and plays an important role in regulating the totipotency of early embryos [66]. Activation of ZSCAN4 is associated with the cellular response to DNA damage. Its expression is sensitive to agents that damage DNA, thereby playing an important role in maintaining genome stability in the early stages of development [67]. As a target gene of DUX4, ZSCAN4, which is essential for telomere extension in embryonic stem cells, is also activated in numerous cancers that express DUX4, potentially enhancing tumor replicative capacity [68]. Specifically, ZSCAN4 is known for its role in maintaining telomeres and responding to DNA damage by regulating telomere lengthening and chromatin remodeling, thereby increasing the replication potential of cancer cells [69]. In cancers such as sarcoma and lymphoma, where DUX4 is re-expressed due to genetic rearrangements or mutations, ZSCAN4 expression results in telomere repair. Typically, telomere shortening restricts cell division, but ZSCAN4 reactivation helps overcome these limitations and promotes tumor cell longevity and continuous division [70]. In these cancers, ZSCAN4 activation enhances cell cycle processes and prevents replicative senescence, thereby promoting tumor growth and metastasis.

In the process of embryogenesis, DUX4 has been shown to activate retrotransposons such as HERV-L [71]. Upon activation, these reverse loci initiate transcription processes by providing cis-regulatory elements, non-coding RNA, or functional proteins. This process contributes to the establishment of a pluripotent state in the early embryo. This pluripotent state is critical for the differentiation of totipotent cells into diverse cell lineages during early embryonic cleavage. The activation of retrotransposons by DUX4 is part of broader transcriptional reprogramming, a process that is essential for establishing embryonic pluripotency and early developmental potential [1]. Retrotransposons can activate their own promoters to regulate gene expression and transcribe long non-coding RNAs. This activity can lead to suppression of tumor suppressor gene function. DUX4 expression in cancer cells induces an early embryonic program that promotes metastasis while simultaneously suppressing immune responses, thereby enhancing tumor growth [45,72]. Collectively, retrotransposon activation not only contributes to the initiation of early embryonic programs but may also facilitate immune evasion, a common feature of many cancers. DUX4-mediated retrotransposon activation during early development serves as a precursor to the mechanisms underlying genomic instability in cancer, wherein similar retrotransposon dysregulation contributes substantially to cancer pathogenesis [73].

During the early stage of development, DUX4 plays a key role in chromatin modification that is involved in ZGA. DUX4 recruits specific chromatin remodeling factors and transcription regulatory factors to targeted genomic loci. This process ensures proper gene activation necessary for embryonic development. Histone acetyltransferases (HATs), such as P300 and CBP, are necessary for this chromatin remodeling process [10,51]. Histone acetyltransferases (HATs), particularly p300/CBP complexes, are essential for ZGA because their acetylation of specific histone tail residues promotes chromatin decompaction, thereby facilitating RNA Polymerase II access to gene promoters and subsequent transcriptional initiation. The histone acetylation mechanism is prominently observed during early embryogenesis, wherein the p300/CBP coactivator complex catalyzes acetylation at the promoters of critical developmental genes, particularly those under DUX4 regulation. In fact, DUX4 recruits p300 to specific target genes, such as ZSCAN4, a gene crucial for early embryonic development. The molecular interaction between DUX4 and p300 enhances histone acetylation at these genomic loci, thereby maintaining the chromatin in an accessible state for transcriptional activity [74,75]. In some cancers caused by DUX4 rearrangement, this chromatin remodeling promotes tumor progression by increasing the ability of cancer cells to replicate. Specifically, within oncogenic chromatin domains, histone acetylation mediated by P300 and CBP can increase transcription activity and maintain tumor cell proliferation and survival. Furthermore, the recruitment of p300 by DUX4 at sites such as *Zscan4* leads to global acetylation changes, particularly at the H3K27 mark, which plays a critical role in gene activation [10].

In preimplantation embryos, DUX4 plays a role in the establishment of the totipotent state, which is characteristic of embryos in the cleavage stage [1]. DUX4 activates a genetic program to maintain the two basic characteristics of stem cells, namely, self-renewal and pluripotency [76]. This role is essential for proper embryonic development and is tightly regulated during early developmental stages to ensure correct cell lineage differentiation. In carcinogenesis, DUX4 expression leads to the reactivation of a developmental program that promotes cancer cell stemness. Recent studies demonstrate that DUX4 expression in various cancers, including sarcoma and leukemia, induces a metastable early embryonic totipotent program. This induction increases the ability of cancer cells to proliferate and promotes their resistance to differentiation. DUX4 modulates the expression of multiple downstream target genes, inducing a stem cell-like state that enables cancer cells to evade differentiation signals while maintaining proliferative capacity. Furthermore, DUX4 interacts with key transcriptional regulators, particularly histone acetyltransferases p300/CBP, facilitating chromatin remodeling essential for stemness maintenance in both neoplastic cells and early embryos [74]. This chromatin remodeling enables the sustained expression of genes critical for stem cell identity and pluripotency, thereby conferring resistance to differentiation-inducing signals.

As summarized in Table 1, which delineates the parallels between neoplastic and embryonic cells, DUX4 functions as a molecular bridge connecting embryogenesis and oncogenesis through its regulation of cell proliferation, immune evasion, gene activation, chromatin modifications, retrotransposon activation, and pluripotency maintenance.

## 6. Clinical Implications of DUX4-Induced Embryonic Features

The reactivation of DUX4 in cancer cells has significant clinical implications, particularly concerning immune evasion and tumor progression. Understanding these implications will open avenues for therapeutic interventions that exploit the embryonic features induced by DUX4 re-expression [65]. DUX4 reactivation is associated with immune evasion mechanisms, notably the suppression of MHC class I expression, which impairs antigen presentation and facilitates tumor escape from immune surveillance. This suppression compromises the effectiveness of immune checkpoint inhibitors, as evidenced by reduced progression-free and overall survival in patients with DUX4-expressing tumors [52,65]. The embryonic program induced by DUX4 reactivation presents potential therapeutic targets. For instance, the transient expression of DUX4 in cancer cells induces a metastable early embryonic stem cell program, including the activation of zygotic gene activation (ZGA) and eight-cell-like (8C-like) transcriptional programs. These programs are characterized by the expression of genes involved in early lineage specification, such as trophectoderm and epithelial/mesenchymal transition markers, which may contribute to tumor progression and metastasis [45].

In this review, we position DUX4 as a unique “developmental hijacker” that orchestrates the reactivation of cleavage-stage transcriptional programs in cancer. This novel framework emphasizes that DUX4 may serve as a master regulator at the nexus of embryonic and cancer cell states, with capabilities that distinguish it from other developmental factors. While stemness regulators, such as OCT4, SOX2, and NANOG, primarily maintain pluripotency, DUX4 uniquely initiates totipotency programs and activates retrotransposons, representing a more primitive developmental state [1]. Further work by Smith et al. demonstrated that DUX4 expression in cancer cells induces a metastable early embryonic totipotent program, which fundamentally alters cellular identity and promotes aggressive phenotypes [45]. Unlike other stemness factors that primarily act through conventional promoters, DUX4 has the distinctive ability to activate both embryonic genes and retrotransposons, creating a more comprehensive reprogramming effect [49]. Epigenetic drugs that modulate stemness and aggressiveness can also produce similar effects in cancers. For example, valproic acid (VPA), a histone deacetylase inhibitor, has been shown to affect osteosarcoma progression by reprogramming cancer cells toward a more stem-like state through epigenetic modifications similar to those mediated by DUX4. Additionally, VPA treatment in osteosarcoma cell lines increases the expression of certain DUX4 target genes, suggesting a mechanistic overlap [77].

Correspondingly, therapeutic strategies targeting DUX4-mediated pathways are multifaceted, and targeting the pathways activated by DUX4 could disrupt these embryonic programs, potentially hindering tumor growth and dissemination. For instance, targeting these aberrant epigenetic modifications caused by DUX4 represents a promising therapeutic strategy. Although DUX4 expression induces a metastable early embryonic stem cell program and suppresses antigen presentation, contributing to immune evasion, targeting the epigenetic regulators involved in this reprogramming may be possible to reverse the immunosuppressive tumor microenvironment [45]. Additionally, inhibiting DUX4 transcriptional activity may be another approach. For instance, in CIC-DUX4 sarcomas, the fusion protein’s oncogenic activity depends on the histone acetyltransferases P300/CBP. Inhibitors like iP300w have demonstrated efficacy in suppressing CIC-DUX4 transcriptional activity and reversing associated histone acetylation, leading to tumor growth inhibition in preclinical models [78]. Additionally, the identification of DUX4 expression as a biomarker for immune evasion suggests its utility in stratifying patients for immunotherapy responsiveness. Counteracting the downstream effects of DUX4 on immune evasion mechanisms may also be a promising avenue. Despite the DUX4-mediated suppression of MHC class I, therapies aimed at enhancing antigen presentation or modulating the tumor microenvironment to promote immune cell infiltration could mitigate DUX4-induced immune suppression [52]. Taken together, therapeutic strategies may include restoring epigenetic modifications caused by DUX4, inhibiting DUX4 transcriptional activity, or counteracting its downstream effects on immune evasion mechanisms. Therapeutic strategies that target these embryonic features or restore immune recognition hold promise for improving cancer treatment outcomes. Therefore, further research into the mechanisms of DUX4-mediated immune suppression and embryonic program activation will be crucial in developing effective interventions.

It should also be borne in mind that other key transcription factor families may exhibit similar dual roles in both developmental and cancer contexts. MYC family transcription factors, critical for embryonic development, exhibit oncogenic properties when dysregulated, driving proliferation through similar molecular mechanisms in both contexts [79]. HOX genes, fundamental for embryonic patterning and organ development, show altered expression patterns in multiple cancer types, with both tumor-promoting and tumor-suppressive roles depending on the specific HOX gene and cellular context [80]. The EMT-inducing transcription factors TWIST and SNAIL, essential for embryonic morphogenesis, are frequently reactivated in cancers to promote invasion and metastasis through similar cellular reprogramming mechanisms [81]. Additionally, FOXM1, crucial for cell cycle progression during embryogenesis, is overexpressed in numerous cancer types, promoting genomic instability and proliferation [82]. These reinforce the conceptual framework that cancer frequently hijacks embryonic transcriptional programs, not just through DUX4 but through multiple transcription factor networks that normally operate during development. This conceptual framework would provide us with a more comprehensive understanding of the developmental origins of cancer, contextualizing our DUX4 findings within a broader paradigm of embryonic program reactivation in tumorigenesis.

## 7. Conclusions

The similarity between embryogenesis and tumorigenesis reveals a common biological process that supports development and cancer. DUX4, acting as a key transcription factor for early embryonic development, functions analogously in embryogenesis and tumorigenesis. The findings on the relationship between embryogenesis and carcinogenesis pave a new way for understanding cancer biology, highlighting the importance of developmental pathways in the behavior of normal and malignant cells. Furthermore, the common DUX4 regulation mechanism may reveal potential targets for cancer treatment, especially in cancers caused by abnormal embryonic program reactivation. Looking ahead, it can be said that targeted therapy that takes advantage of this developmental fragility can open new avenues for more effective cancer treatment, especially ways of reprogramming cancer cells into a more differentiated and less aggressive state.

## Figures and Tables

**Table 1:** The role of DUX4 in linking embryogenesis to oncogenesis.

Shared Features of Embryonic and Cancer Cells	Role of DUX4	Reference
Uncontrolled proliferation	Promotes proliferation and blocks differentiation	[62,63]
Immune evasion	Suppresses MHC I, aiding immune escape	[52,64,65]
Undergo early developmental processes	Triggers cleavage-stage transcription	[66,67,68,69,70]
Retrotransposon activation	Activates HERV-L	[1,45,71,72,73]
Chromatin remodeling	Recruits p300/CBP to drive gene activation	[10,51,74,75]
Pluripotency and stemness	Induces totipotent-like state and self-renewal programs	[1,74,76]

## Data Availability

No new data were created or analyzed in this study.
